# Effect of the soil type on the microbiome in the rhizosphere of field-grown lettuce

**DOI:** 10.3389/fmicb.2014.00144

**Published:** 2014-04-08

**Authors:** Susanne Schreiter, Guo-Chun Ding, Holger Heuer, Günter Neumann, Martin Sandmann, Rita Grosch, Siegfried Kropf, Kornelia Smalla

**Affiliations:** ^1^Institute for Epidemiology and Pathogen Diagnostics, Julius Kühn-InstitutBraunschweig, Germany; ^2^Department of Plant Health, Leibniz Institute of Vegetable and Ornamental Crops Großbeeren/Erfurt e.V.Großbeeren, Germany; ^3^College of Resources and Environmental Sciences, China Agricultural UniversityBeijing, China; ^4^Institute of Crop Science (340h), Hohenheim UniversityStuttgart, Germany; ^5^Department for Biometrics und Medical Informatics, Otto von Guericke UniversityMagdeburg, Germany

**Keywords:** *Lactuca sativa*, bacterial communities, 16S rRNA gene analysis, DGGE, pyrosequencing, rhizosphere responders

## Abstract

The complex and enormous diversity of microorganisms associated with plant roots is important for plant health and growth and is shaped by numerous factors. This study aimed to unravel the effects of the soil type on bacterial communities in the rhizosphere of field-grown lettuce. We used an experimental plot system with three different soil types that were stored at the same site for 10 years under the same agricultural management to reveal differences directly linked to the soil type and not influenced by other factors such as climate or cropping history. Bulk soil and rhizosphere samples were collected 3 and 7 weeks after planting. The analysis of 16S rRNA gene fragments amplified from total community DNA by denaturing gradient gel electrophoresis and pyrosequencing revealed soil type dependent differences in the bacterial community structure of the bulk soils and the corresponding rhizospheres. The rhizosphere effect differed depending on the soil type and the plant growth developmental stage. Despite the soil type dependent differences in the bacterial community composition several genera such as *Sphingomonas, Rhizobium, Pseudomonas*, and *Variovorax* were significantly increased in the rhizosphere of lettuce grown in all three soils. The number of rhizosphere responders was highest 3 weeks after planting. Interestingly, in the soil with the highest numbers of responders the highest shoot dry weights were observed. Heatmap analysis revealed that many dominant operational taxonomic units were shared among rhizosphere samples of lettuce grown in diluvial sand, alluvial loam, and loess loam and that only a subset was increased in relative abundance in the rhizosphere compared to the corresponding bulk soil. The findings of the study provide insights into the effect of soil types on the rhizosphere microbiome of lettuce.

## Introduction

Plants influence soil microorganisms in the vicinity of their roots through their root architecture, exudates, and mucilage (Bais et al., 2006; Badri and Vivanco, 2009). The so-called rhizosphere effect was already recognized in the beginning of the 20th century by Hiltner (1904). Not only the available nutrients released by the plant but also changes in the pH and redox gradients are assumed to shape the composition of microbial communities in the rhizosphere (Schmidt et al., 2011). Microorganisms which profit from the chemical changes in the vicinity of the roots and utilize these compounds increase in abundance and typically show enhanced metabolic activity. Obstacles to study rhizosphere microbial communities are manifold ranging from sampling the rhizosphere to limitations of traditional cultivation-based methods and resolution level of 16S rRNA gene-based methods. The rhizosphere effect was comprehensively studied by means of molecular fingerprints based on 16S rRNA gene fragments amplified from total community (TC) DNA of bulk soil and rhizosphere (reviewed by Berg and Smalla, 2009). While bulk soils were typically characterized by a high number of faint bands indicating a high evenness of many equally abundant populations, the rhizosphere fingerprints display several intense bands indicating populations with increased abundance in the vicinity of the roots (Smalla et al., 2001; Costa et al., 2006a). When different plant species or cultivars were grown at the same field sites, different fingerprint methods revealed a plant species or even a cultivar-dependent composition of the bacterial communities in the rhizosphere (Smalla et al., 2001; Schmalenberger and Tebbe, 2002; Weinert et al., 2009, 2011). The latter was typically less pronounced. Although it was clear that the soil from which plants select their microbiome must play an important role, the evaluation of the extent to which the soil type influences the microbial community was difficult to assess under field conditions as not only the soil characteristics but also the climate, the cropping history or the agricultural management are assumed to influence the soil microbiome (Costa et al., 2006b, 2007). Therefore, studies investigating the same crops grown at different locations could only report on the effect of the sites. Costa et al. (2006b, 2007) could show that the site was the overriding factor although for *Actinobacteria* or *Pseudomonas* similar populations were enriched in the rhizosphere of strawberries at different locations. Through pyrosequencing of 16S rRNA gene fragments amplified from TC-DNA of *Arabidopsis thaliana* grown in different soils under greenhouse conditions both Bulgarelli et al. (2012) and Lundberg et al. (2012) provided insights into the effects of the soil type on the bacterial community composition in the rhizosphere. However, the effect of the soil type on the microbial community composition in bulk soil and in the rhizosphere has never been studied under field conditions. The importance of the plant microbiome has only recently been recognized and was proposed as the second genome of plants (Berendsen et al., 2012). The fact that many recent studies revealed that the plant microbiome is of great importance for plant growth and health triggered the idea to include the microbiome as an essential part in plant breeding programs (Berendsen et al., 2012). In the present study, we hypothesized that different soil types characterized by different physicochemical properties harbor different microbial communities. The soil type dependent microbial community composition as well as the root exudation patterns and architecture studied by Neumann et al. (2014) might determine the composition of microbial communities in the rhizosphere. We used an experimental plot system with three soil types stored at the same site for 10 years under the same agricultural management to reveal differences directly linked to the soil type and not influenced by other factors like local climate or cropping history under field conditions. In order to investigate to what extent the soil type or the plant determine the bacterial community composition in the rhizosphere, lettuce plants were grown in four replicate plots per soil type. Bulk soil and lettuce rhizosphere were sampled at two time points 3 and 7 weeks after planting (3WAP and 7WAP) and analyzed by denaturing gradient gel electrophoresis (DGGE) and pyrosequencing of 16S rRNA gene fragments amplified from TC-DNA.

## Materials and methods

### Field experiment

#### Experimental design

The field experiment was performed in a unique experimental plot system at the Leibniz Institute of Vegetable and Ornamental Crops (Großbeeren, Germany, 52° 33′ N, 13° 22′ E) to evaluate the effect of soil types on bacterial communities in the rhizosphere and in bulk soil. The experimental system included three soils of different origin in separate blocks: Arenic-Luvisol with less silty sand and 5.5% clay (diluvial sand, DS), Gleyic-Fluvisol with heavy sandy loam and 27.5% clay (alluvial loam, AL), and Luvic-Phaeozem with medium content of clayey silt and 17.2% clay (loess loam, LL) (Rühlmann and Ruppel, 2005). Each block consisted of 24 plots of 2 × 2 m in size and 0.75 m depth (Table S1). Previous crops from 2000 to 2009 were pumpkin, nasturtium, pumpkin, amaranth, wheat, wheat, pumpkin, nasturtium, wheat, and wheat. Lettuce (cultivar “Tizian”) was selected as a model plant in our experiment. Seeds were germinated in a seedling tray containing the respective soil types at 12°C for 48 h and further cultivated under greenhouse conditions at approximately 20/15°C (day/night). All seedlings were watered daily to maintain the soil moisture and fertilized weekly (0.2% Wuxal TOP N, Wilhelm Haug GmbH & Co. KG, Düsseldorf, Germany). Lettuce seedlings pregrown in the same soils were planted at the three- to four-leaf stage (BBCH 13-14) in the experimental system. Each plot included six rows with a within-row and intra-row distance of 30 cm between lettuce plants (36 plants per plot). Four replicate plots were established for each treatment and soil type. Overhead irrigation was applied based on the irrigation computer program “BEREST” (Gutezeit et al., 1993). Input variables for the irrigation program were the daily soil water content in the rooted soil layer using the water holding capacity of the soil, the plant growth stage, and the potential evapotranspiration (Table S2). Irrigation decisions were made on the basis of the calculated soil water content and the expected evapotranspiration and precipitation of the next five days. The temperature (reflectometer PT100b1/3 DIN, Messtechnik Geraberg GmbH, Germany) and the matric potential (tensiometer T22968, transmitter ES 1075, bambach GbR Tensio-Technik, Geisenheim, Germany) were recorded in 10 cm soil depth during the field experiment in four replicates for each soil type (Table S3, Figures S1, S2). One day before planting fertilizer was added based on a chemical analysis of each soil type (Table S1). Each soil type was adjusted to 168 mg/100 g N by fertilizer (Kalkamon, 27% N, TDG mbH Lommatzsch, Germany) to exclude effects by different N contents on lettuce growth. Soil samples for chemical analysis and characterization of soil parameters were taken one week before planting (three random replicates per soil type). Soil analysis was done by the Agricultural Tests and Research Institutions Association (VdLUFA, Germany) according to standard protocols. Lettuce plants were finally harvested by hand 7WAP (BBCH 49) to obtain lettuce shoot dry weight measured for each plant at harvest. The data of lettuce dry weight were parametrically analyzed after ANOVA using Dunnett's procedure with *P* ≤ 0.05 with the STATISTICA program (StatSoft Inc., Tulsa, OK, USA).

#### Sampling and DNA extraction

Bulk soil and rhizosphere samples were collected before planting lettuce into the field as well as 3WAP and 7WAP. Ten cores (10 cm of top soil; 2 cm core diameter) of bulk soil were randomly taken from each plot and mixed by sieving (mesh size 2 mm). From these approximately 200 g soil a subset of approximately 2 g was collected in a 2 ml Eppendorf tube and stored at −80°C until DNA extraction. For the rhizosphere samples the complete root systems of three plants per plot were combined as a composite sample after removing loosely adhering soil by vigorous shaking. Microbial cells were extracted from the samples as follows: plant roots were cut into pieces of approximately 1 cm length using scissors, carefully mixed and treated by a Stomacher 400 Circulator (Seward Ltd, Worthing, UK) for 30 s at high speed after adding 15 ml sterile 0.3% NaCl to 5 g root pieces. After centrifugation at 500 *g* for 2 min the supernatant was collected and the resulting pellet was re-suspended, transferred to the Stomacher bag with root pieces and exposed to another Stomacher treatment after adding 15 ml sterile 0.3% NaCl. This step was repeated one more time and the combined supernatants of three Stomacher blending steps (45 ml) were centrifuged at 10.000 *g* for 30 min to obtain the microbial pellet. The supernatant of this processing step was discarded and the pellet was re-suspended in the remaining solution, transferred to a 2 ml reaction tube and centrifuged at 14.000 *g* for 20 min. The pellets were stored at −80°C.

TC-DNA was extracted from 0.5 g of bulk soil or the microbial pellets obtained from 5 g of roots with tightly adhering soil using the FastDNA SPIN Kit for Soil® (MP Biomedicals, Heidelberg, Germany) after a harsh lysis step as described by the manufacturer. The TC-DNA was purified with GENECLEAN SPIN Kit® (MP Biomedicals, Heidelberg, Germany) according to the manufacturer's instructions and was 1:10 diluted with 10 mM Tris HCl pH 8.0 before use.

#### DGGE analysis of 16S rRNA gene fragments amplified from TC-DNA

16S rRNA gene fragments were PCR-amplified from TC-DNA of bulk soil and rhizosphere samples using the bacterial primers F984-GC and R1378 as described by Heuer et al. (1997). The PCR products were analyzed by DGGE. The gradient of the DGGE gel was performed as described in Weinert et al. (2009) and the electrophoresis conditions as well as the silver staining procedure were done according to Heuer et al. (2001).

#### Analysis of the DGGE fingerprints

Bacterial DGGE community fingerprints were evaluated with GELCOMPAR II version 6.5 (Applied Maths, Sint-Martens-Latem, Belgium). The gel images were normalized and the background was subtracted according to the spectral analysis of each gel. For establishing the similarity matrix a curve based method was chosen. The fingerprints were grouped according to their similarity using the hierarchical cluster method UPGMA (unweighted pairwise grouping method using arithmetic means) based on Pearson correlation coefficient for each pair of lanes. The Pearson similarity matrices were analyzed by means of the permutation test calculating the *d*-value from the average overall correlation coefficients within the groups minus the average overall correlation coefficients between samples from different groups as suggested by Kropf et al. (2004) to test the significant differences in community composition between the soil types, rhizosphere, and bulk soil at two sampling times.

#### Pyrosequencing and statistical analysis

16S rRNA gene fragments amplified from TC-DNA of rhizosphere and bulk soil samples collected 3WAP and 7WAP were analyzed by barcoded pyrosequencing for all replicates. The PCR reaction and the sequencing of the hypervariable V3-V4 region of the 16S rRNA gene was performed at the Biotechnology Innovation Center (BIOCANT, Cantanhede, Portugal) using the primers 338F and 802R (Huse et al., 2008; Vaz-Moreira et al., 2011) which were fused to the 454 A and B adaptors, respectively. Sequencing was performed on a 454 Genome Sequencer FLX platform according to standard protocols (Roche—454 Life Sciences, Branford, CT, USA).

The analysis of the pyrosequencing data was done according to Ding et al. (2012a). Briefly, only those sequences matching the barcode and primer were selected for BLASTN analysis against a SILVA 16S rRNA gene database to truncate the unpaired regions for each sequence. Low quality sequences or chimera resulted in a short alignment which was subsequently filtered out. Only those sequences with a length of more than 200 bp were included in the analysis. Operational taxonomic units (OTUs) were generated with the following steps: sequences were assigned to OTUs (defined at 97% sequences similarity) with the program Mothur 1.21. software (Schloss et al., 2009) and the Naïve Bayesian Classifier (Wang et al., 2007) was used to classify the sequences. The OTU assignment and the classification of each sequence were loaded into a MySQL-data base for producing the taxonomic OTU report. Statistical analysis of the OTU report was done with the Tukey's honest significance test and visualization of the result was performed with R 2.15 (http://www.r-project.org). The Pearson similarity matrices based on relative abundance of the OTUs were analyzed by means of the permutation test calculating the *d*-value as described for DGGE by Kropf et al. (2004).

For the comparison of the community composition between samples the number *n* of sequences for each OTU was divided by the total number of sequences *N* from the sample and transformed by log(*n/N* * 1000 + 1). The transformed data were used to analyze the effect of soil type, habitat (rhizosphere or bulk soil), and their interaction by a modified principal components test according to Ding et al. (2012b) in the rotation test version.

Pyrosequencing data were deposited at the NCBI Sequence Read Archive under the study accession number SRP029944.

## Results

### Soil characteristics, cultivation conditions, and lettuce growth

The three soil types displayed striking differences not only in the mineral composition but also in pH, total C, N, P and their content in metals and trace elements (Table S1). Furthermore, the average temperature in the 10 cm top soil measured during the vegetation of lettuce was significantly different for AL and LL (LSD test, *P* ≤ 0.05). On average a temperature of 16.3°C (range between 9.6 and 26.9°C) was recorded in AL and of 15.9°C (9.1–26.4°C) in LL. No significant differences were detected between average soil temperature of 16.2°C (range between 9.1 and 26.4°C) in DS and the other soil types. The highest day/night temperatures were measured within the first two weeks of lettuce growth in all three soils (Figure S1). In contrast the volumetric soil water content (VWC) varied significantly between all soils (Table S3). The lowest percent VWC was recorded in DS whereas the highest VWC was observed in AL (Figure S2, Tables S2, S3). On average a percent VWC of 15.7% was calculated in DS, of 29.4% in AL, and of 24.3% in LL soil. Furthermore, a mean daily global radiation of 3.28 kWh m^−2^ was measured during the growth period of lettuce.

A total of 120 lettuce plants per soil type grown in four plots with 30 plants per plot were harvested 7WAP. Lettuce plants grown in AL showed with 31.8 g per plant the highest shoot dry weight on average compared to lettuce plants grown in DS (24.4 g/plant) and in LL soil (20.9 g/plant). The dry weight of lettuce harvested from AL soil was significantly higher compared to the shoot dry weight of plants grown in the other two soil types according to Dunnett's procedure (*P* ≤ 0.05) while no significant differences were observed for lettuce grown in DS or LL soil.

### DGGE analysis of 16S rRNA gene fragments amplified from total community DNA revealed

#### Soil type dependent bacterial community composition in bulk soil

Bacterial community DGGE fingerprints of bulk soil samples taken 3WAP and 7WAP from all three soil types displayed that some of the bands were soil type specific, while most of the bands were shared among all soil types (Figures S3, S4). The permutation test of the bacterial community fingerprints revealed statistically significant differences between the three bulk soils (DS-AL; DS-LL; AL-LL) at both sampling times (Table 1). Higher dissimilarities (*d*-values) between bacterial community fingerprints, in particular of DS-AL and DS-LL were observed 3WAP compared to 7WAP. The lowest differences were observed between AL and LL soil fingerprints at both sampling times (*d*-value 3WAP 22.4, 7WAP 20.1) (Table 1) indicating that the bacterial community composition of AL and LL bulk soil were more similar to each other compared to DS bulk soil.

**Table 1:** **Soil type dependent differences of bacterial communities in bulk soil and rhizosphere**.

**Method**	**Figure**	**Sampling time**	**Differences in the bulk soil**		
			**DS-AL**	**DS-LL**	**AL-LL**
DGGE	S3	3WAP	41.8^*^	44.9^*^	22.4^*^
	S4	7WAP	30.4^*^	28.8^*^	20.1^*^
Pyrosequencing		3WAP	28.3^*^	38.2^*^	17.3^*^
		7WAP	23.5^*^	29.9^*^	16.7^*^
			**Differences between bulk soil and rhizosphere**		
			**DS**	**AL**	**LL**
DGGE	S3	3WAP	18.1^*^	28.1^*^	23.7^*^
	S4	7WAP	23.1^*^	30.2^*^	20.3^*^
Pyrosequencing		3WAP	37.9^*^	42.2^*^	32.7^*^
		7WAP	42.6^*^	30.5^*^	32.3^*^
			**Differences in the rhizosphere**		
			**DS-AL**	**DS-LL**	**AL-LL**
DGGE	S3	3WAP	22.8^*^	29.2^*^	29.0^*^
	S4	7WAP	19.8^*^	32.3^*^	26.7^*^
Pyrosequencing		3WAP	22.0^*^	33.0^*^	29.2^*^
		7WAP	20.2^*^	46.5^*^	30.8^*^

*Percent dissimilarity (d-value) based on DGGE fingerprints and pyrosequncing of Bacteria from bulk soil and rhizosphere of lettuce (cv. “Tizian”) grown in three soils (DS, diluvial sand; AL, alluvial loam; LL, loess loam) of an experimental unit at the same field site. Samples were collected 3 and 7 weeks after planting (3WAP, 7WAP). The asterisks indicate significant differences (P ≤ 0.05) between the soil types determined by a permutation test*.

#### Soil type dependent rhizosphere effect

Compared to the corresponding bulk soil fingerprints a number of bands with stronger intensity was typically detected in the lettuce rhizosphere fingerprints (Figures S3, S4), indicating that some populations were enriched in the rhizosphere. Significant differences in the rhizosphere and the corresponding bulk soil bacterial community fingerprints were detected for all soils but the extent of this rhizosphere effect differed depending on the soil type and the sampling time. Measures for the extent of the rhizosphere effect were the *d*-values obtained after permutation test analysis. At both sampling times the highest *d*-values were observed for AL soil (*d*-values: 3WAP 28.1, 7WAP 30.2) indicating that the strongest rhizosphere effect was observed in AL soil. The lowest *d*-values were observed for DS soil 3WAP and 7WAP for LL soil (Table 1).

#### Soil type dependent rhizosphere community composition

Cluster analysis based on the Pearson correlation indices showed that the bulk and rhizosphere fingerprints formed separate clusters for each of the soil types. Interestingly, at both time points the rhizosphere fingerprints of AL clustered with the DS rhizosphere, once again indicating a strong shift of bacterial community in the rhizosphere of lettuce grown in AL soil (Figures S5, S6). Statistical analysis confirmed that the bacterial communities in the rhizosphere of lettuce grown in the three soil types were significantly different at both time points. Indeed the lowest *d*-values were obtained for DS-AL at both time points.

#### Shifts in the bacterial community composition with plant development

Bacterial community fingerprints of rhizosphere samples taken before transplanting, 3WAP and 7WAP from DS, AL, and LL indicated changes in the bacterial community composition over time (Figure S7). While many bands were detected at all time points, some bands with changes in band intensity depending on the plant growth developmental stage were identified. Typically these changes were seen for all four replicates analyzed.

### Pyrosequencing

The 16S rRNA gene amplicons from TC-DNA of 47 samples (the same TC-DNA used for DGGE analysis) were sequenced and altogether 249,350 sequences with a sequence length of more than 200 bp were obtained. The sequences from DS rhizosphere replicate b taken 7WAP had to be excluded from the analysis because the number of obtained sequences was unusually low and treated as an outlier.

A total of 23 phyla, 49 classes, 55 orders, 145 families, 421 genera, and 28,650 OTUs were obtained. Dominant phyla were defined as phyla with more than 1% relative abundance. The phyla *Proteobacteria*, *Actinobacteria*, *Firmicutes*, *Acidobacteria*, and *Bacteroidetes* were dominant in bulk soil as well as in the rhizosphere of all three soil types (Table 2). In the bulk soil 3WAP the highest relative abundance was observed for *Proteobacteria*, followed by the *Actinobacteria*, *Firmicutes*, *Acidobacteria*, and *Bacteroidetes* in all three soil types (Table 2). Compared to bulk soil, the relative abundance of *Proteobacteria* was significantly enhanced in the rhizosphere of lettuce grown in all three soil types at both sampling times. The strongest increase in relative abundance was observed for DS and AL soil. Especially *Gammaproteobacteria* were enriched in DS soil, whereas *Betaproteobacteria* were enriched in AL and LL soil with up to a four times increase in AL 7WAP. In contrast, in comparison to the corresponding bulk soil the relative abundance of *Actinobacteria* was significantly decreased in all rhizospheres. The relative abundance of *Firmicutes* was lower in the rhizosphere of lettuce grown in AL and DS compared to the corresponding bulk soil but remained nearly unchanged for LL soil (Table 2). The relative abundance of the *Acidobacteria* decreased in the rhizosphere of lettuce compared to the corresponding bulk soil (except for AL 3WAP), while the relative abundance of *Bacteroidetes* increased in the rhizosphere compared to the corresponding bulk soil with the strongest increase in the rhizosphere of lettuce grown in AL of up to two and a half times higher at 3WAP. This strong enrichment could not be detected 7WAP. In the LL soil even a decrease in the rhizosphere compared to the corresponding bulk soil was observed (Table 2).

**Table 2:** **Relative abundance of phyla in bulk soil and rhizosphere**.

**Sampling time**	**Phylum**	**Class**	**DS**		**AL**		**LL**	
			**Bulk soil**	**Rhizosphere**	**Bulk soil**	**Rhizosphere**	**Bulk soil**	**Rhizosphere**
3WAP	*Proteobacteria*		29.7 ± 1	50.6 ± 1^*^	29.0 ± 1	45.7 ± 1^*^	32.1 ± 1	44.4 ± 3^*^
7WAP	*Proteobacteria*		30.7 ± 1	50.3 ± 2^*^	30.2 ± 1	42.7 ± 3^*^	33.0 ± 4	44.3 ± 10^*^
3WAP	*Proteobacteria*	*Alphaproteobacteria*	15.8 ± 1	30.7 ± 2^*^	15.7 ± 1	27.0 ± 1^*^	17.4 ± 1	27.1 ± 3^*^
7WAP	*Proteobacteria*	*Alphaproteobacteria*	17.9 ± 2	33.3 ± 3^*^	17.8 ± 1	21.8 ± 1^*^	17.9 ± 1	28.4 ± 8^*^
3WAP	*Proteobacteria*	*Betaproteobacteria*	5.3 ± 0	10.0 ± 1^*^	3.6 ± 0	8.2 ± 1^*^	3.7 ± 0	6.1 ± 0^*^
7WAP	*Proteobacteria*	*Betaproteobacteria*	4.3 ± 2	8.4 ± 1^*^	2.3 ± 0	10.1 ± 4^*^	2.8 ± 1	7.5 ± 3^*^
3WAP	*Proteobacteria*	*Deltaproteobacteria*	4.6 ± 0^*^	2.2 ± 0	4.6 ± 1^*^	2.5 ± 0	4.2 ± 0^*^	2.7 ± 0
7WAP	*Proteobacteria*	*Deltaproteobacteria*	4.5 ± 1^*^	2.1 ± 0	5.1 ± 1^*^	2.9 ± 0	4.5 ± 0^*^	2.6 ± 1
3WAP	*Proteobacteria*	*Gammaproteobacteria*	2.6 ± 0	5.9 ± 1^*^	3.6 ± 0	6.6 ± 1^*^	4.7 ± 0	6.5 ± 1^*^
7WAP	*Proteobacteria*	*Gammaproteobacteria*	2.4 ± 0	5.1 ± 0^*^	3.6 ± 0	6.6 ± 2^*^	6.0 ± 3^*^	4.0 ± 1
3WAP	*Actinobacteria*		26.4 ± 1^*^	12.7 ± 1	29.7 ± 2^*^	11.7 ± 1	29.7 ± 1^*^	16.7 ± 3
7WAP	*Actinobacteria*		19.0 ± 3^*^	11.7 ± 1	23.9 ± 2^*^	15.1 ± 2	22.8 ± 2^*^	16.1 ± 3
3WAP	*Firmicutes*		13.5 ± 1^*^	7.3 ± 1	11.8 ± 1^*^	6.9 ± 0	10.6 ± 1	12.0 ± 3^*^
7WAP	*Firmicutes*		16.3 ± 3^*^	7.7 ± 0	12.0 ± 1^*^	7.8 ± 3	12.0 ± 3	14.2 ± 6^*^
3WAP	*Acidobacteria*		10.3 ± 0^*^	7.0 ± 0	9.4 ± 1	9.1 ± 1	7.6 ± 1^*^	5.6 ± 2
7WAP	*Acidobacteria*		12.4 ± 1^*^	6.8 ± 1	11.3 ± 1^*^	7.7 ± 1	8.7 ± 1^*^	6.6 ± 0
3WAP	*Bacteroidetes*		4.7 ± 1	8.7 ± 1^*^	5.2 ± 1	12.9 ± 1^*^	5.6 ± 0	8.4 ± 1^*^
7WAP	*Bacteroidetes*		5.5 ± 1	6.8 ± 1	6.6 ± 1	10.2 ± 3^*^	7.7 ± 2^*^	5.7 ± 2

*Relative abundance [%] of phyla in the rhizosphere of lettuce (cv. “Tizian”) compared to the bulk soil 3 and 7 weeks after planting (3WAP, 7WAP) based on pyrosequencing data. Lettuce plants were grown in three soils (diluvial sand, DS; alluvial loam, AL and loess loam, LL) in an experimental unit at the same field site. The asterisks indicate significant differences (P ≤ 0.05) between the bulk soil and corresponding rhizosphere samples identified by Tukey's honest test under a generalized linear model via logistic function for binomial data*.

### Soil type-dependent bacterial community composition in the rhizosphere and in bulk soil

**Figure 1:**
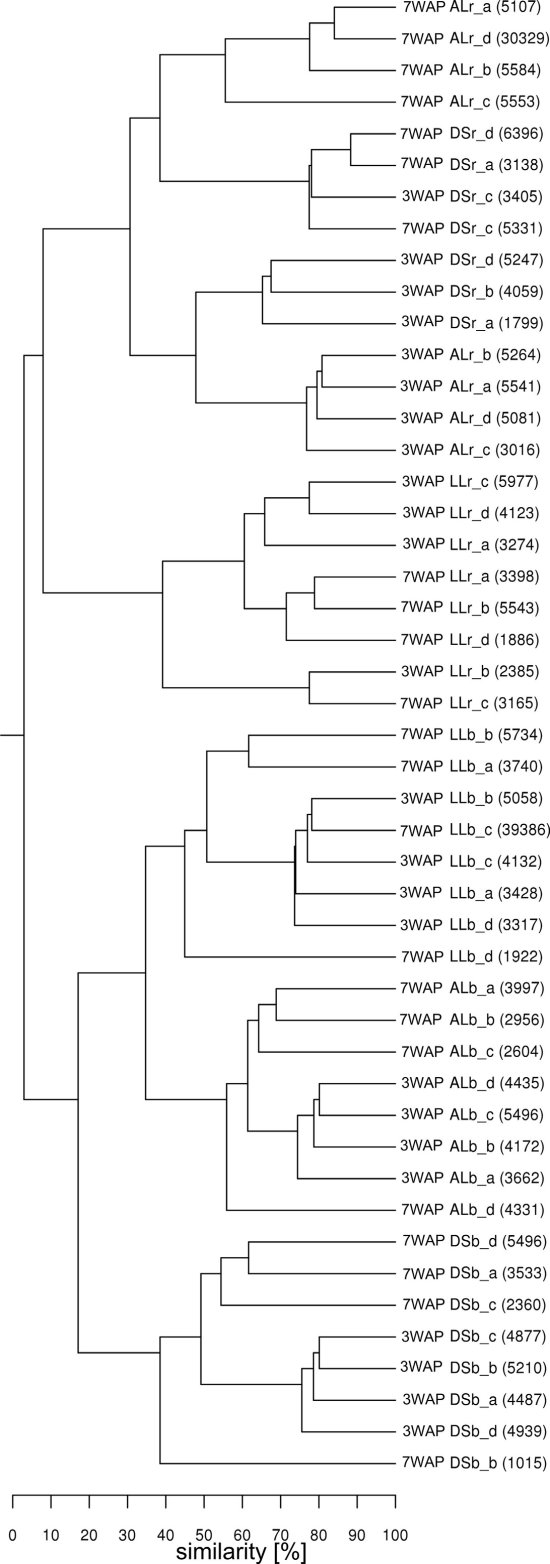
**Cluster analysis of *bacterial* communities from rhizosphere and bulk soil samples**. Samples from rhizosphere of lettuce and the corresponding bulk soil of DS, AL, and LL soil were taken at 3 and 7 weeks after planting (3WAP, 7WAP). Similarities between samples were calculated as the Pearson correlation of the relative abundance of OTUs based on pyrosequencing data. Numbers in brackets indicate the number of sequences for the sample; r: rhizosphere samples; b: bulk soil samples; a–d: replicates.

**Figure 2:**
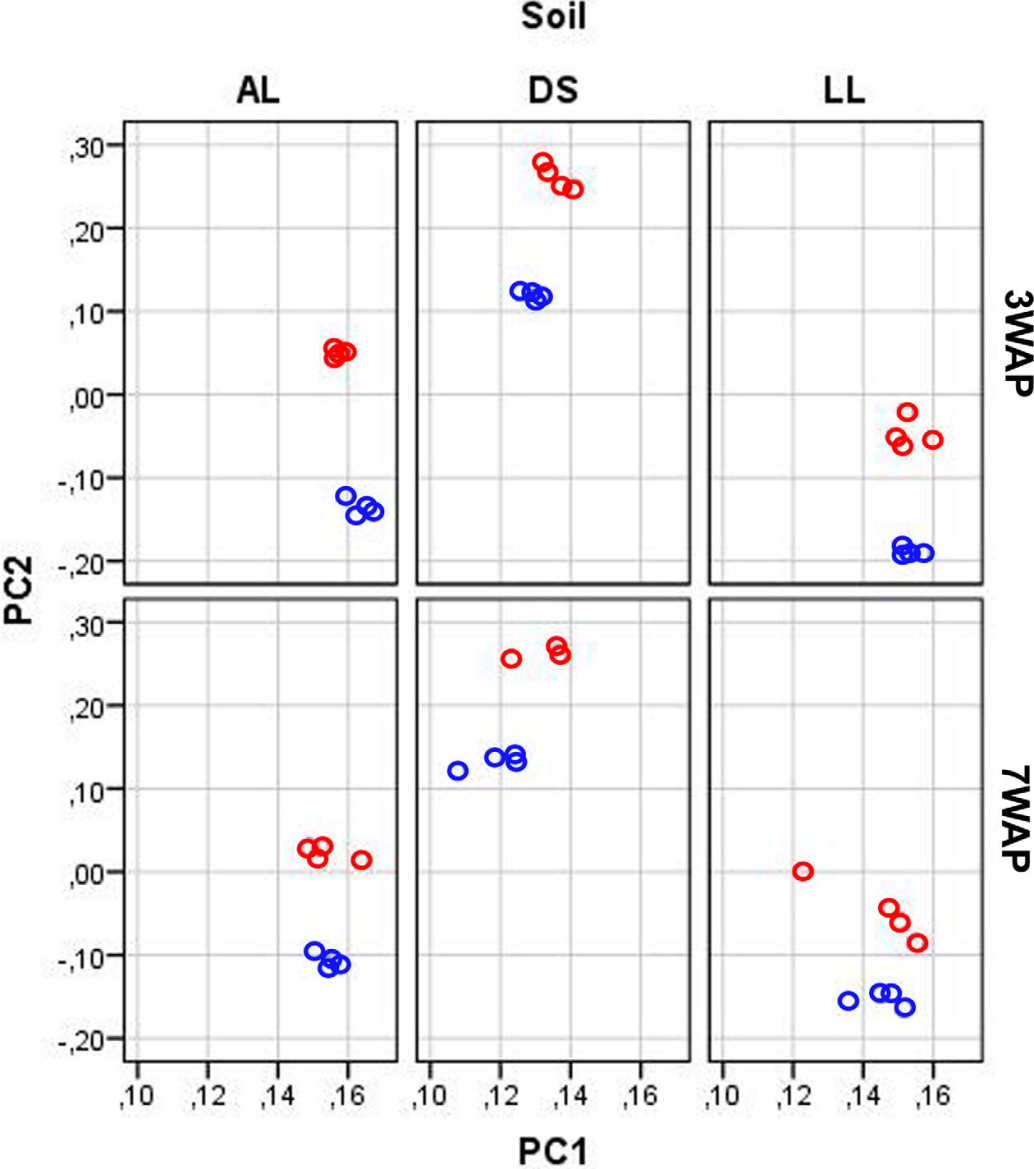
**Principal component analysis of the bacterial community composition according to pyrosequencing data**. The relative abundance of OTUs (log transformed) in samples from rhizosphere (red circles) and bulk soil (blue circles) taken 3 and 7 weeks after planting (3WAP, 7WAP) of lettuce in soils DS, AL, and LL were compared. The first and second principal components are shown which explained 47 and 9% of the total variance, respectively. The data were analyzed together, but for clarity separated plots for each soil type and sampling are shown.

UPGMA analysis was based on relative abundance of all bacterial OTUs (≥ 97% sequence identity) obtained for bulk soil and rhizosphere samples from both sampling times (Figure 1). Two main clusters were obtained clearly separating the fingerprints bulk soil and rhizosphere samples. Within the bulk soil cluster three soil type dependent clusters were formed (Figure 1). Samples from both sampling times clustered together for each soil type with a trend to sub-clusters for samples from 3WAP and 7WAP. Typically a higher similarity of all four replicates per soil type was observed 3WAP compared to 7WAP. The AL and LL bulk soils clustered, indicating that the bacterial communities in the two loamy soils showed a higher similarity to each other compared to the bacterial community of DS bulk soil which formed a separate cluster. This was confirmed by the *d*-values which were lowest for the AL-LL soil comparison (Table 1). However, in the rhizosphere the bacterial communities of DS and AL became more similar and formed a joint cluster with sub-clusters according to sampling time and soil type (Figure 1). This was supported by the lowest *d*-value for the comparison of DS-AL rhizosphere. The rhizosphere of lettuce grown in LL soil clustered separately with nearly no influence of the sampling time. The lowest *d*-value was observed 3WAP for the comparison LL_bulk soil_-LL_rhizosphere_. The *d*-values for the comparison between bulk soil and rhizosphere were in the range of the differences between bulk soils and were highest for AL 3WAP and DS 7WAP, respectively (Table 1). Two-factorial multivariate analysis of variance by a modified principal components test revealed highly significant effects of the soil type and of the habitat (rhizosphere or bulk soil) on the community composition for both sampling times (*P* < 0.001). Also the interaction effect was significant (*P* = 0.0001) which means that the rhizosphere effect was soil type dependent (Figure 2). The sampling time had an effect on the community composition, but the trends were the same for both samplings (Figure 2). Rhizosphere and bulk soil communities of each soil type were clearly separated in principal component analysis, and the communities of both habitats were separated according to the soil type. Communities from soils AL and LL were more similar to each other than to DS for rhizosphere and bulk soil at both samplings. In soil LL the communities from rhizosphere and bulk soil were more closely related than in the other two soils (Figure 2).

### Soil type dependent and independent responders in the lettuce rhizosphere

The discriminative taxa between the soil types were identified by Tukey's honest significance test under a generalized linear model via logistic function for binomial data.

Twenty-four genera were significantly increased in abundance in the rhizosphere compared to the corresponding bulk soil at least in one of the soils 3WAP (Table 3). The highest number of rhizosphere responders was detected in AL (15) followed by DS (13) and LL (11) soils. The relative abundances of the genera *Sphingomonas, Rhizobium, Pseudomonas, Variovorax*, and *Flavobacterium* were enriched in the rhizosphere of lettuce grown in all three soils 3WAP. At this sampling time, the genus *Rhizobium* showed the strongest relative enrichment in the rhizosphere in DS and AL soil, in the LL soil *Rhizobium* was not detected in the bulk soil but increased to 1.2% in the rhizosphere. Several rhizosphere responders were soil type specific. Furthermore, 3WAP the highest number of soil type specific rhizosphere responders was observed in AL (*Adheribacter*, *Burkholderia*, *Chitinophaga*, *Flavisolibacter*, *Pedobacter, Phenylobacterium*, *Pontibacter*, TM7) followed by DS (*Brevundimonas, Dyadobacter, Hydrogenophaga, Methylibium*, *Rubellimicrobium*) and LL soil (*Paenibacillus*). Several rhizosphere responders were only detected in two of the three soils. Thus, *Acidovorax*, *Devosia*, and *Ramlibacter* were only significantly enriched in DS and LL rhizospheres and *Acinetobacter* and *Novosphingobium* in AL and LL rhizospheres (Table 3). The number of rhizosphere responders identified 7WAP was lower compared to 3WAP as only 12 genera were found to be significantly increased in abundance in the rhizosphere of lettuce compared to the corresponding bulk soil (Table 4). The highest number of rhizosphere responders was detected in AL (9) and LL (8) followed by DS rhizosphere (7). Again, *Sphingomonas, Rhizobium*, and *Variovorax* were enriched in all three soil types. In addition *Acidovorax* and *Methylophilus* were enriched in the rhizosphere of lettuce grown in all three soil types (Table 4). *Pseudomonas* was only enriched in the DS and AL soils 7WAP, while *Burkholderia* was detected as rhizosphere responders in AL and LL soils. *Flavobacterium* and *Acinetobacter* were specific rhizosphere responders for AL soils, *Mesorhizobium* was specific for DS soil while *Caulobacter* and *Devosia* were rhizosphere responders in LL soil.

**Table 3:** **Enriched genera in the rhizosphere of lettuce 3 weeks after planting**.

**Genus**	**DS**		**AL**		**LL**	
	**Bulk soil**	**Rhizosphere**	**Bulk soil**	**Rhizosphere**	**Bulk soil**	**Rhizosphere**
*Acidovorax*	0.0 ± 0	1.5 ± 0^*^	0.0 ± 0	0.2 ± 0	0.0 ± 0	0.6 ± 0^*^
*Acinetobacter*	0.0 ± 0	0.2 ± 0	0.0 ± 0	0.7 ± 1^*^	0.0 ± 0	0.7 ± 1^*^
*Adhaeribacter*	0.2 ± 0	0.2 ± 0	0.6 ± 0	1 ± 0^*^	0.5 ± 0	0.5 ± 0
*Brevundimonas*	0.1 ± 0	0.3 ± 0^*^	0.0 ± 0	0.1 ± 0	0.0 ± 0	0.2 ± 0
*Burkholderia*	0.0 ± 0	0.1 ± 0	0.0 ± 0	0.8 ± 0^*^	0.0 ± 0	0.0 ± 0
*Chitinophaga*	0.1 ± 0	0.0 ± 0	0.0 ± 0	0.3 ± 0^*^	0.0 ± 0	0.1 ± 0
*Devosia*	0.2 ± 0	0.6 ± 0^*^	0.0 ± 0	0.3 ± 0	0.0 ± 0	0.5 ± 0^*^
*Dyadobacter*	0.0 ± 0	0.4 ± 0^*^	0.0 ± 0	0.1 ± 0	0.0 ± 0	0.3 ± 0
*Flavisolibacter*	0.1 ± 0	0.1 ± 0	0.2 ± 0	0.5 ± 0^*^	0.2 ± 0	0.1 ± 0
***Flavobacterium***	0.0 ± 0	0.3 ± 0^*^	0.1 ± 0	0.3 ± 0^*^	0.1 ± 0	0.5 ± 0^*^
*Hydrogenophaga*	0.0 ± 0	0.5 ± 0^*^	0.0 ± 0	0.2 ± 0	0 ± 0	0.3 ± 0
*Methylibium*	0.1 ± 0	0.5 ± 0^*^	0.2 ± 0	0.4 ± 0	0.2 ± 0	0.1 ± 0
*Novosphingobium*	0.0 ± 0	0.1 ± 0	0.1 ± 0	0.4 ± 0^*^	0.1 ± 0	0.4 ± 0^*^
*Paenibacillus*	2.1 ± 0	1.8 ± 0	1.7 ± 0	1.5 ± 0	1.9 ± 0	3.6 ± 1^*^
*Pedobacter*	0.2 ± 0	0.3 ± 0	0.0 ± 0	0.4 ± 0^*^	0.2 ± 0	0.3 ± 0
*Phenylobacterium*	0.2 ± 0	0.5 ± 0	0.2 ± 0	0.4 ± 0^*^	0.1 ± 0	0.3 ± 0
*Pontibacter*	0.0 ± 0	0.0 ± 0	0.3 ± 0	0.7 ± 0^*^	1 ± 0	1.4 ± 0
***Pseudomonas***	0.1 ± 0	0.4 ± 0^*^	0.1 ± 0	0.7 ± 0^*^	0.2 ± 0	0.8 ± 0^*^
*Ramlibacter*	0.1 ± 0	0.5 ± 0^*^	0.1 ± 0	0.3 ± 0	0.1 ± 0	0.3 ± 0^*^
***Rhizobium***	0.1 ± 0	1.5 ± 0^*^	0.1 ± 0	2.0 ± 0^*^	0.0 ± 0	1.2 ± 0^*^
*Rubellimicrobium*	0.2 ± 0	0.7 ± 0^*^	0.1 ± 0	0.1 ± 0	0.0 ± 0	0.1 ± 0
***Sphingomonas***	2.2 ± 0	8.2 ± 1^*^	2.2 ± 0	8.9 ± 1^*^	1.7 ± 0	4.7 ± 1^*^
TM7_genera_incertae_sedis	0.1 ± 0	0.3 ± 0	0.1 ± 0	0.4 ± 0^*^	0.1 ± 0	0.4 ± 0
***Variovorax***	0.0 ± 0	0.3 ± 0^*^	0.1 ± 0	0.6 ± 0^*^	0.0 ± 0	0.5 ± 0^*^

*The relative abundance of genera in the rhizosphere of lettuce, grown in the three soil types (diluvial sand, DS; alluvial loam, AL and loess loam, LL), was compared with the corresponding bulk soil. Percent abundance of genera ±SD is shown. The asterisks indicate significantly enriched genera in the rhizosphere identified by Tukey's honest test under a generalized linear model via logistic function for binomial data (P ≤ 0.05). Genera in bold letters indicate enrichment in all three soil types*.

**Table 4:** **Enriched genera in the rhizosphere of lettuce 7 weeks after planting**.

**Genus**	**DS**		**AL**		**LL**	
	**Bulk soil**	**Rhizosphere**	**Bulk soil**	**Rhizosphere**	**Bulk soil**	**Rhizosphere**
***Acidovorax***	0.0 ± 0	1.0 ± 0^*^	0.0 ± 0	0.6 ± 0^*^	0.0 ± 0	0.5 ± 0^*^
*Acinetobacter*	0.1 ± 0	0.1 ± 0	0.0 ± 0	0.9 ± 1^*^	0.3 ± 0	0.0 ± 0
*Burkholderia*	0.0 ± 0	0.1 ± 0	0.0 ± 0	2.0 ± 1^*^	0.0 ± 0	0.6 ± 0^*^
*Caulobacter*	0.0 ± 0	0.4 ± 0	0.0 ± 0	0.2 ± 0	0.0 ± 0	0.2 ± 0^*^
*Devosia*	0.1 ± 0	0.3 ± 0	0.1 ± 0	0.1 ± 0	0.0 ± 0	0.4 ± 0^*^
*Flavobacterium*	0.1 ± 0	0.2 ± 0	0.3 ± 0	1.1 ± 1^*^	0.6 ± 0	0.5 ± 0
*Mesorhizobium*	0.1 ± 0	0.6 ± 0^*^	0.2 ± 0	0.3 ± 0	0.3 ± 0	0.2 ± 0
***Methylophilus***	0.0 ± 0	0.4 ± 0^*^	0.0 ± 0	0.6 ± 0^*^	0.0 ± 0	0.4 ± 0^*^
*Pseudomonas*	0.3 ± 1	0.7 ± 0^*^	0.3 ± 0	1.7 ± 1^*^	2.0 ± 2	0.5 ± 0
***Rhizobium***	0.1 ± 0	1.4 ± 0^*^	0.1 ± 0	0.7 ± 0^*^	0.2 ± 0	0.9 ± 0^*^
***Sphingomonas***	3.2 ± 1	14.0 ± 3^*^	3.0 ± 0	5.8 ± 0^*^	2.2 ± 0	4.1 ± 0^*^
***Variovorax***	0.0 ± 0	0.6 ± 0^*^	0.1 ± 0	0.5 ± 0^*^	0.2 ± 0	0.6 ± 1^*^

*The relative abundance of genera in the rhizosphere of lettuce, grown in the three soil types (diluvial sand, DS; alluvial loam, AL and loess loam, LL), was compared with the corresponding bulk soil. Percent abundance of genera ±SD is shown. The asterisks indicate significantly enriched genera in the rhizosphere identified by Tukey's honest test under a generalized linear model via logistic function for binomial data (P ≤ 0.05). Genera in bold letters indicate enrichment in all three soil types*.

### Only a subset of dominant OTUs was enriched in the rhizosphere of lettuce

**Figure 3:**
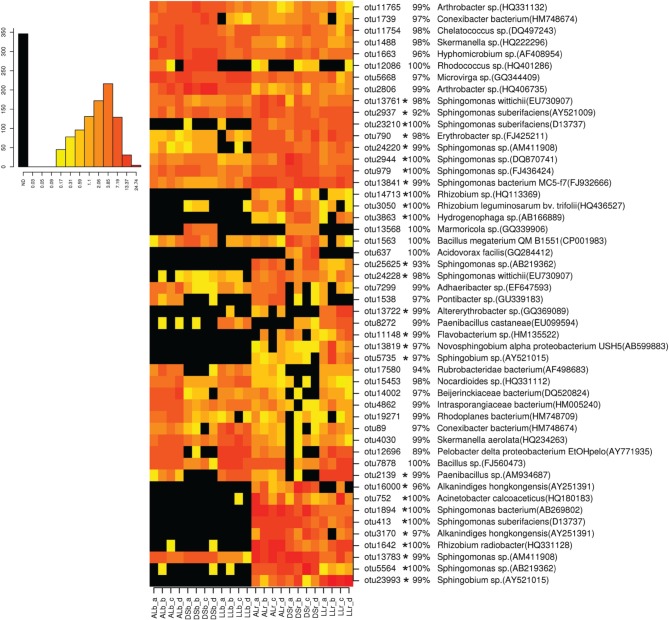
**Relative abundance of the most dominant OTUs detected 3 weeks after planting**. The heatmap indicates differences in the relative abundances of OTUs in the bulk soil and rhizosphere from lettuce, and between soil types DS, AL, and LL. The vertical columns represent one sample, horizontal rows depict OTUs. The color code grades from black (not detected) over yellow (low abundance), orange (medium abundance) to red (high abundance). Numbers in brackets indicate the number of the NCBI GenBank accession that was most similar to the OTU representative sequence. A strong increase in abundance was indicated by not detecting the OTU in the bulk soil (black) or only in one or two samples present (yellow), raising to a high abundance in the rhizosphere which was indicated by orange to red color. Asterisks indicate a significantly increased abundance of that OTU in the rhizosphere compared to bulk soil.

**Figure 4:**
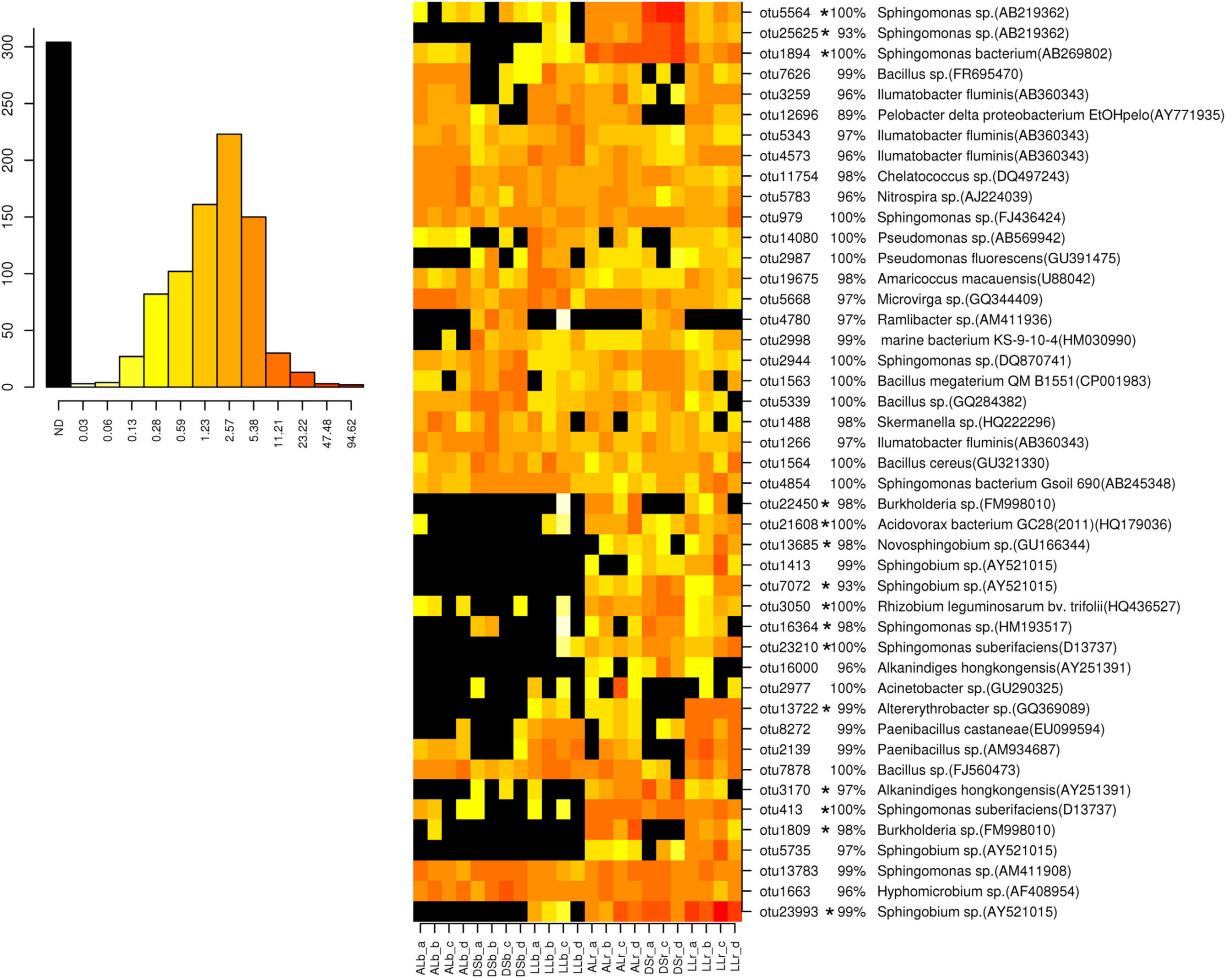
**Relative abundance of the most dominant OTUs detected 7 weeks after planting**. The heatmap indicates differences in the relative abundances of OTUs in the bulk soil and rhizosphere from lettuce, and between soil types DS, AL, and LL. The vertical columns represent one sample, horizontal rows depict OTU. The color code grades from black (not detected) over yellow (low abundance), orange (medium abundance) to red (high abundance). Numbers in brackets indicate the number of the NCBI GenBank accession that was most similar to the OTU representative sequence. A strong increase in abundance was indicated by not detecting the OTU in the bulk soil (black) or only in one or two samples present (yellow), raising to a high abundance in the rhizosphere which was indicated by orange to red color. Asterisks indicate a significantly increased abundance of that OTU in the rhizosphere compared to bulk soil.

For analysis at the OTU level the OTUs with an abundance of more than 0.5% were selected (Figures 3, 4). Thirty-four of the dominant OTUs in the rhizosphere taken 3WAP were affiliated to *Proteobacteria* (34/50) with the vast majority being *Alphaproteobacteria* (28/34) followed by the phyla *Actinobacteria* (9/50), *Firmicutes* (4/50), and *Bacteriodetes* (3/50). At the later sampling time (7WAP) 32 of the 45 most dominant OTUs were affiliated also to the *Proteobacteria* (32/45) with the majority being *Alphaproteobacteria* (23/32) followed by the phyla *Firmicutes* (7/45) and *Actinobacteria* (4/45). The relative abundances of 27 of the 50 dominant OTUs were significantly enriched in the rhizosphere 3WAP whereas 7WAP less OTUs were enriched.

Strong rhizosphere responders 3WAP displayed a high sequence identity with isolates *Sphingomonas* sp. (OTU 1894), *Sphingomonas suberifaciens* (OTU 413), *Alkanindiges hongkonggensis* (OTU 3170), *Rhizobium radiobacter* (OTU 1642), and *Sphingobium* sp. (OTU 23993) (Figure 3). Strong responders at the later sampling time (7WAP) were *Sphingomonas* sp. (OTU 25625), *Burkholderia* sp. (OTU 22450), *Novosphingobium* sp. (OTU 13685), *Alkanindiges hongkonggensis* (OTU 16000), and *Sphingobium* sp. (OTU 5735) (Figure 4).

Several OTUs showed a soil type specific occurrence 3WAP, e.g., *Rubrobacteridae* (OTU 17580), *Nocardioides* sp. (OTU 15453), *Bacillus* sp. (OTU 7878), and *Paenibacillus* (OTU 2139) were less abundant in the DS soil compared to AL and LL soil (Figure 3). OTU 13568 which displayed 100% sequence identity to *Marmoricola* sp. was detected only in DS rhizosphere and bulk soil. The OTU 637 identified as *Acidovorax facilis* was only detected in DS rhizosphere samples (Figure 3).

Several soil type specific OTUs were detected among the dominant OTUs 7WAP, e.g., *Pelobacter* (OTU 12696) which was less abundant in DS soil and *Ramlibacter* sp. (OTU 4780) which was only detected in DS soil (Figure 4).

## Discussion

Our study showed that different soil types exposed to identical cropping history and agricultural management for about 10 years and identical climatic conditions for more than 30 years still displayed a soil type dependent bacterial diversity. Although the soil type was identified as a major factor shaping composition of microbial communities in the rhizosphere in many previous studies, this study showed the importance of the soil type under field conditions. The same main dominant phyla were detected in the three soils, but significant differences in the bacterial community composition among the soils were detected by both DGGE and pyrosequencing analyses of 16S rRNA gene fragments amplified from TC-DNA. UPGMA analyses of DGGE fingerprints and of OTUs as well as the principal component analyses indicated a higher similarity of AL and LL soils compared to DS soil which corresponded to the more similar soil characteristics of the AL and LL soils for many parameters including pH, electric conductivity, fine grained particles, total, and organic C, N, P, and all metal ions measured (Table S1). All these parameters were lower in the DS soil. Recently, Kuramae et al. (2012) reported that several soil bacterial taxa were strongly correlated to physicochemical soil characteristics.

The experimental set-up allowed for the first time to determine the effect of the soil type on the lettuce rhizosphere bacterial community composition under field conditions. However, we could not pinpoint specific soil properties responsible for these differences as the soil properties were determined before the field experiment. The statistical analysis of both DGGE and pyrosequencing data sets revealed significant differences in the bacterial community composition between the rhizosphere of the three soils. In particular, for AL and DS the bacterial community composition became more similar in the rhizosphere compared to bulk soil (Table 1, Figure 1, Figure S3), while for soil LL the rhizosphere community was more similar to that of the corresponding bulk soil than to the rhizosphere communities of AL and DS (Figure 2). The data indicate that depending on the soil type the rhizosphere was differently shaped by lettuce growth. Soil type dependent composition of bacterial communities in the rhizosphere of *Arabidopsis thaliana* grown under greenhouse conditions in different soils was also observed by Bulgarelli et al. (2012) and Lundberg et al. (2012). Although the same phyla were reported in the rhizosphere of different plants (Uroz et al., 2010; Bulgarelli et al., 2012; Lundberg et al., 2012; Dohrmann et al., 2013) their relative abundances substantially differed.

Remarkably, in all three soil types similar shifts in the relative abundance of the major phyla were detected in response to lettuce growth which might be attributed to the root exudates and deposits of lettuce. Root exudates were investigated in parallel with the same lettuce cultivar “Tizian” planted in DS, AL, or LL soil in minirhizotron systems which were equipped with root observation windows (Neumann et al., 2014). The GC-MS analysis revealed qualitatively similar root exudates which quantitatively differed depending on the soil type. Thus, the relative abundance of OTUs affiliated to *Proteobacteria* almost doubled in DS, AL, and LL soil at both sampling times indicating that bacterial populations with copiotrophic lifestyle belonging to the *Proteobacteria* were able to utilize the exudates and deposits provided by lettuce roots. The proportion of most proteobacterial classes was increased in the rhizosphere in all three soil types except for *Deltaproteobacteria*. An increased abundance of *Proteobacteria* in the rhizosphere compared to bulk soils was reported in several recent studies based on 16S rRNA amplicon sequencing (Bulgarelli et al., 2012; Lundberg et al., 2012). The proportion of *Actinobacteria* and *Firmicutes* in the rhizosphere of lettuce decreased in all three soil types. Several OTUs that belonged to these phyla were similarly abundant in both rhizosphere and bulk soil, while an enrichment in the rhizosphere was not observed (Figures 3, 4). For a long time it has been assumed that microbial community composition and function in the rhizosphere are tightly linked to the root exudation patterns (Brimecombe et al., 2001) and that changes in the exudates composition result in dramatic changes of the soil microbial community composition. Root exudation patterns were assumed to be affected by the plant growth developmental stage (Baudoin et al., 2002) and several biotic and abiotic factors. Root secretion of some proteins was shown to be changed with plant development or when challenged with pathogenic or symbiotic bacteria. But only recently, Chaparro et al. (2013) could show how root-secreted primary and secondary plant metabolites change during plant growth development, and pyrosequencing of mRNA revealed a tight link with microbial functions involved in metabolism of the root exudates. In the present study changes in the bacterial community composition likely related to plant development were observed by both methods employed for 16S rRNA amplicon analysis. Shifts in the bacterial community composition in lettuce rhizosphere during plant growth were previously also reported by Adesina et al. (2009) and Chowdhury et al. (2013). Both the *d*-values obtained from the statistical analyses of DGGE fingerprints and pyrosequencing data set as well as the number of so-called responders to the lettuce root exudates which was higher 3WAP compared to 7WAP indicated that the rhizosphere effect was stronger at the earlier time point. Although the root length and exudation patterns of lettuce plants (cultivar “Tizian”) grown in DS, AL, and LL soil under rhizotron conditions differed significantly (Neumann et al., 2014), similar genera were selected in the rhizosphere of lettuce plants of all soil types. Several genera which were significantly enriched in the rhizosphere of lettuce grown in all three soils (Tables 3, 4) were previously reported as degraders of aromatic hydrocarbons or pesticides with aromatic ring structures such as *Sphingomonas*, *Pseudomonas*, and *Variovorax* (Bers et al., 2011; Ding et al., 2012a). Although the abundance of these genera significantly increased in the rhizosphere of lettuce grown in all three soils, the extent of enrichment seemed to be different in particular for the genus *Sphingomonas* that showed a remarkably increased abundance in DS soil followed by AL and LL. The comparative analysis of the relative abundance of the most dominant OTUs in the rhizosphere and in bulk soil showed that enrichment in the rhizosphere seemed to be species- or even strain-dependent as only some of the OTUs affiliated to *Sphingomonas* were strongly enriched (Figure 3). We could previously also demonstrate an enrichment of IncP-1 plasmids in the rhizosphere of lettuce in all three soils which was particularly pronounced for DS soil (Jechalke et al., 2014). IncP-1 plasmids carry frequently genes encoding degradative functions. Thus, it is tempting to speculate that *Sphingomonas* might be the host of these IncP-1 plasmids. Also strains of other genera such as *Burkholderia*, *Novosphingobium*, and *Acinetobacter* that are possibly involved in the degradation of aromatic ring structures were identified as rhizosphere responders 7WAP (Figure 4). This nicely correlated with the detection of benzoic acid in the root exudates collected from lettuce plants grown in DS, AL, and LL soil under rhizotron conditions (Neumann et al., 2014). Benzoic acid was also previously reported in root exudates of lettuce grown in hydroponics (Lee et al., 2006). In the rhizotron experiment performed by Neumann et al. (2014) with lettuce grown in DS, AL, and LL soils, a strong effect of the soil type was detected not only on the quantitative composition of root exudates but also on root biomass production and root length. Total root length in AL, mainly represented by fine roots of 0–0.4 mm diameter (70% of total root length) was about two and a half times higher as compared with DS, and even four times higher than in LL soil. In general, mainly quantitative differences in the exudate profiles were detected (Neumann et al., 2014) which might explain that several similar responders to lettuce growth were identified based on the pyrosequencing data set (Tables 3, 4; Figures 3, 4). Another member of the *Alphaproteobacteria*, the genus *Rhizobium*, known for specific interactions with host plants was found to be enriched in the rhizosphere of lettuce independent from the soil type and at both sampling times. Interestingly, the genus *Acidovorax* was found to be enriched in the rhizosphere independently from the soil type (Tables 3, 4). Several species belonging to the genus *Acidovorax* are known plant pathogens (Thiele et al., 2012). However, the differentiation between *Variovorax*, often involved in the degradation of aromatic compounds, and *Acidovorax* is complicated based on 16S rRNA gene sequence analysis (Bers et al., 2011). The identification of similar but also soil type specific responders in different soils likely provides insights into populations triggered by root exudates. Whether lettuce plant exudates deliberately increase the abundance of particular populations or whether the shifts in the bacterial community composition are merely due to the nutrients provided remains to be shown. Our data clearly demonstrated the strong effects of lettuce growth on the bacterial community composition in the rhizosphere in all three soils as revealed by analyzing DGGE and pyrosequencing data. Pyrosequencing confirmed the reduced diversity in the rhizosphere as previously assumed based on DGGE fingerprints and provided insights into the taxonomic affiliation of rhizosphere responders which were shared by all soils or which were specific to particular soil types. DGGE and pyrosequencing data indicated that lettuce grown in AL soil overall had the strongest rhizosphere effect which correlated with the highest root biomass observed in the study by Neumann et al. (2014) and to the highest shoot dry mass observed under field conditions. Remarkably, many dominant OTUs (Figures 3, 4) were detected in all three soil types and differed mainly in their relative abundance which nicely corresponds to the high number of bands shared among the bacterial community fingerprints of all three soils (Figures S3, S4). The heatmap analysis also indicated that several OTUs which were not detected at all in the bulk soil became detectable after their enrichment in the rhizosphere.

Although 16S rRNA gene sequences-based conclusions on potential functions are problematic (Eltlbany et al., 2012), we have noticed that many genera and OTUs were enriched in the rhizosphere known for their involvement in the degradation of aromatic compounds. Similar responders were observed in response to phenanthrene pollution from two soil types as revealed by pyrosequencing of 16S rRNA gene fragments from soil TC-DNA (Ding et al., 2012a). In conclusion, the present study revealed that three different soil types exposed for more than 10 years to the same climatic conditions and cropping history still displayed distinct bacterial community compositions. Pyrosequencing analysis of 16S rRNA gene amplicons largely confirmed the DGGE data but provided more quantitative data and taxonomic information on the bacterial community composition and information on main responders to the lettuce growth at two time points of plant development. The present study showed under field conditions that both the plant as well as the soil type shape the bacterial community composition in the rhizosphere. Several rhizosphere responders were detected independently from the soil type indicating taxa which are likely enriched in the rhizosphere and thus might allow predictions on the soil type dependent rhizosphere competence of inoculant strains.

### Conflict of interest statement

The authors declare that the research was conducted in the absence of any commercial or financial relationships that could be construed as a potential conflict of interest.
