# Supplementary material for: Effect of the soil type on the microbiome in the rhizosphere of field-grown lettuce
Source: Front Microbiol. 2014 Apr 8;5:144. doi: 10.3389/fmicb.2014.00144 (PMC3986527; doi:10.3389/fmicb.2014.00144)

**Supporting material
Effect of the soil type on the microbiome in the rhizosphere of field-grown lettuce**

Susanne Schreiter^1,4^, Guo-Chun Ding^1,2^, Holger Heuer^1^, Günter Neumann^3^, Martin Sandmann^4^, Rita Grosch^4^ , Siegfried Kropf^5^ and Kornelia Smalla^1^

^1^Julius Kühn-Institut, Institute for Epidemiology and Pathogen Diagnostics, Messeweg 11-12, D-38104 Braunschweig, Germany

^2^College of Resources and Environmental Sciences, China Agricultural University, Beijing, People's Republic of China

^3^Universität Hohenheim, Fg. Ernährungsphysiologie der Kulturpflanzen, Fruwirthstr. 20, D-70599 Stuttgart, Germany

^4^Leibniz Institute of Vegetable and Ornamental Crops Großbeeren/Erfurt e.V., Department Plant Health, Theodor-Echtermeyer-Weg 1, D-14979 Großbeeren, Germany

^5^Institute for Biometry and medical informatics, Otto von Guericke University, Leipziger Straße 44, D-39120 Magdeburg, Germany

Key words: *Lactuca* *sativa,* bacterial communities, 16S rRNA gene analysis, DGGE, pyrosequencing, rhizosphere responders

Email of corresponding author: Kornelia.smalla@jki.bund.de

**Table S1 | Soil parameters.** Parameters of soils included in the experimental plot system at the Institute of Vegetable and Ornamental Crops in Großbeeren, Germany. The DS soil (diluvial sand) was described as an Arenic-Luvisol with less silty sand and 5.5% clay (silty sand). The AL soil (alluvial loam) was an Gleyic-Fluvisol, heavy sandy loam with 27.5% clay (sandy loam) and the LL soil (loess loam ) was a Luvic-Phaeozem, a medium clayey silt with 17.2% clay (clayey silt (Rühlmann et al., 2005). Soil parameters were analyzed according to the certified protocols of Agricultural Tests and Research Institutions Association (VdLUFA, Germany).

| Soil type | DS | AL | LL |
| --- | --- | --- | --- |
| pH | 6.1 | 6.7 | 7.3 |
| Conductivity, EC [µS/cm] | 50.0 | 92.6 | 103.5 |
| Bulk density, TRD [g/cm^3^] | 1.5 | 1.3 | 1.4 |
| Clay content (< 2 µm), CC [%] | 5.5 | 27.9 | 17.2 |
| Fine grained particles (< 6.3 µm), FAT [%] | 7.7 | 34.9 | 23.6 |
| Sand particles 200-2000 µm [%] | 35.3 | 30.8 | 5.8 |
| Depositing particles (<20 µm) [%] | 11.5 | 31.6 | 43.4 |
| Total carbon content, Ct [%] | 0.8 | 1.8 | 1.9 |
| Organic carbon content, Corg [%] | 0.9 | 1.8 | 1.8 |
| N content before fertilizer application [mg/100 g] | 71.0 | 159.9 | 168.5 |
| P [mg/100 g] | 25.4 | 46.0 | 26.1 |
| K [mg/100 g] | 12.3 | 26.0 | 30.0 |
| Na [mg/100 g] | 7.6 | 12.2 | 17.1 |
| Mg [mg/100 g] | 5.8 | 16.0 | 14.0 |
| Ca [mg/100 g] | 188 | 611 | 819 |
| Cu [mg/100 g] | 3.6 | 3.6 | 3.2 |
| Fe [mg/100 g] | 545 | 2060 | 1580 |
| Zn [mg/100 g] | 7.6 | 11.2 | 9.2 |

**Table S2** | **Mean parameter estimates for the van Genuchten model**. Based on additional $n=6$ measurements for DS and AL and $n=2$ measurements for LL using the evaporation method (Peters and Durner 2008). The parameters were necessary to compute the volumetric soil water content using the matric potential $h$ in $\mathrm{hPa}$ of the soil, which was provided by hourly tensiometer measurements.

| **Parameter** | **Unit** | **DS** | **AL** | **LL** |
| --- | --- | --- | --- | --- |
| $\alpha$ | $c\boldsymbol{m}^{\boldsymbol{-1}}$ | 0.0232 | 0.5 | 0.0371 |
| $n$ | $-$ | 2.043 | 1.218 | 1.226 |
| $\theta_{r}$ | $cm^{3}/cm^{3}$ | 0.087 | 0.232 | 0 |
| $\theta_{s}$ | $cm^{3}/cm^{3}$ | 0.337 | 0.52 | 0.426 |

The terms $\theta$_r_ describe the residual and $\theta$_s_ the saturated soil water content where $\alpha$ in $cm^{-1}$ and $n$ (dimensionless) are soil specific parameters.

**Table S3 |** **Volumetric soil water content.** Comparisons of the volumetric soil water contents of the soil types AL, DS, and LL, assuming hetroscedasticity in the ANOVA-analysis.

| **Soil types** | **Estimate** | **SE** | **DF** | **P** | **CIl** | **CIu** |
| --- | --- | --- | --- | --- | --- | --- |
| AL-DS | 0.1362 | 0.004565 | 77.5 | <0.0001 | 0.1253 | 0.1471 |
| AL-LL | 0.0503 | 0.004827 | 74.1 | <0.0001 | 0.0388 | 0.0618 |
| DS-LL | -0.0859 | 0.005738 | 93.5 | <0.0001 | -0.0996 | -0.0722 |

(Estimate = difference of least square means, SE = standard error, DF = degree of freedom with adjustment after Kenward and Roger, P = P-value from Tukey test, CIl = 95% lower confidence limit, CIu = 95% upper confidence limit).

**Figure S1** | **Average soil temperature**. Samples were taken in 10 cm top soil of three different soil types (DS, AL and LL soil) measured during the vegetation period day (D) and night (N)of lettuce grown on the experimental unit 6 in Großbeeren, Germany.

**Figure S2** | **Volumetric water content measured during the cultivation of lettuce grown in the experimental unit in Großbeeren, Germany.** Calculated from tensiometer measurements ($15-20 cm$ soil depth), using the van Genuchten model with the parameters from Table S2.

**Figure S3** | ***Bacteria* DGGE fingerprint.** Samples were taken three weeks after planting (3WAP) from DS, AL, and LL bulk soils (b) and the corresponding lettuce rhizosphere (r). M: Marker; letters a-d on top of lanes indicate the four replicates per treatment; arrows marked with letter a indicate soil type specific bands / b: bands present in all three soil types / c: bands with increased intensity in the rhizosphere fingerprints compared to the corresponding bulk soil.


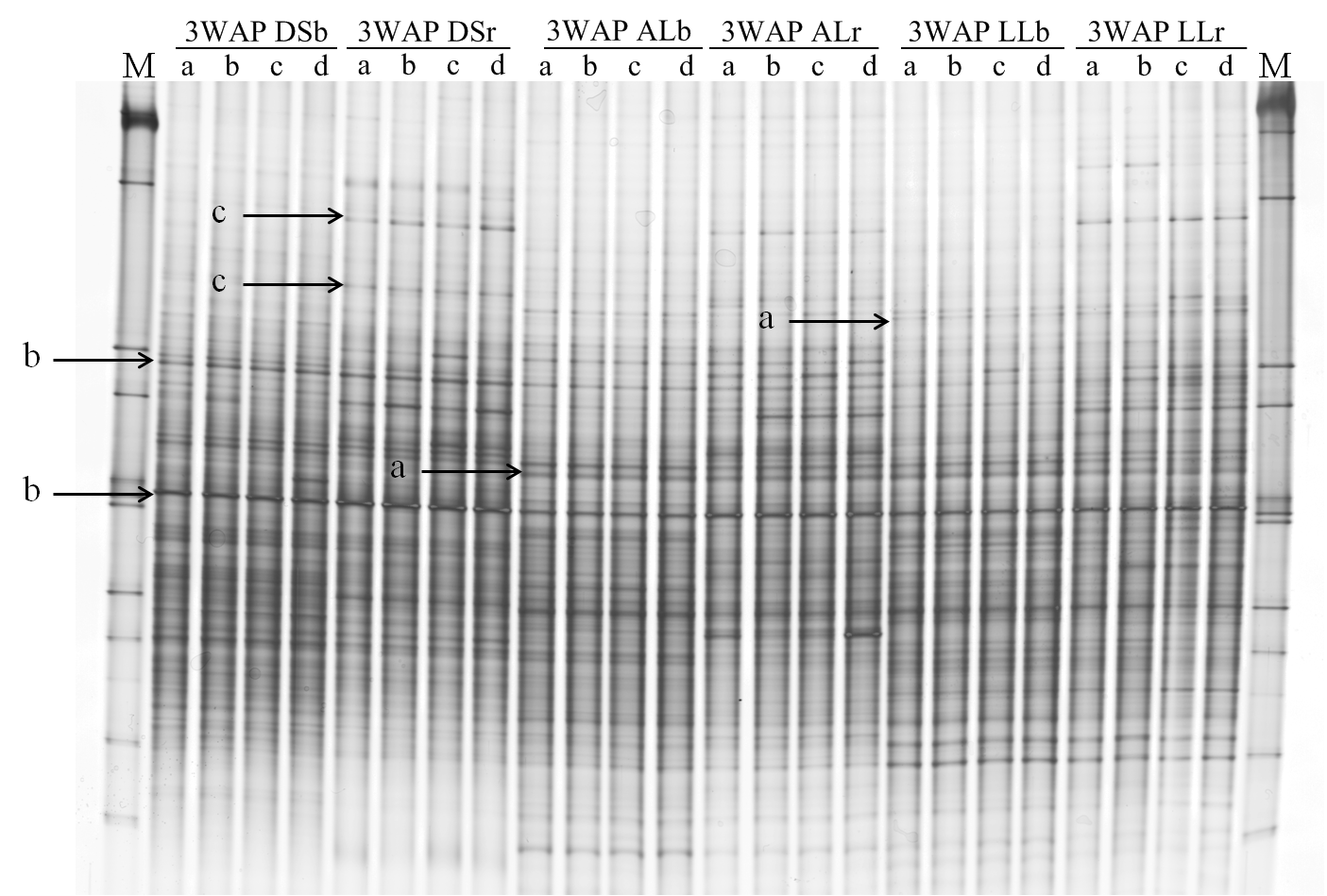


**Figure S4**| ***Bacteria* DGGE fingerprint**. Samples were taken seven weeks after planting (7WAP) from DS, AL, and LL bulk soils (b) and the corresponding lettuce rhizosphere (r). M: Marker; *S.j*.: *Serratia plymuthica* 3Re4-18; *P.j.*: *Pseudomonas jessenii* RU47; letters a-d on top of lanes indicate the four replicates per treatment; arrows marked with letter a indicate soil type specific bands / b: bands present in all three soil types / c: bands with increased intensity in the rhizosphere fingerprints compared to the corresponding bulk soil.
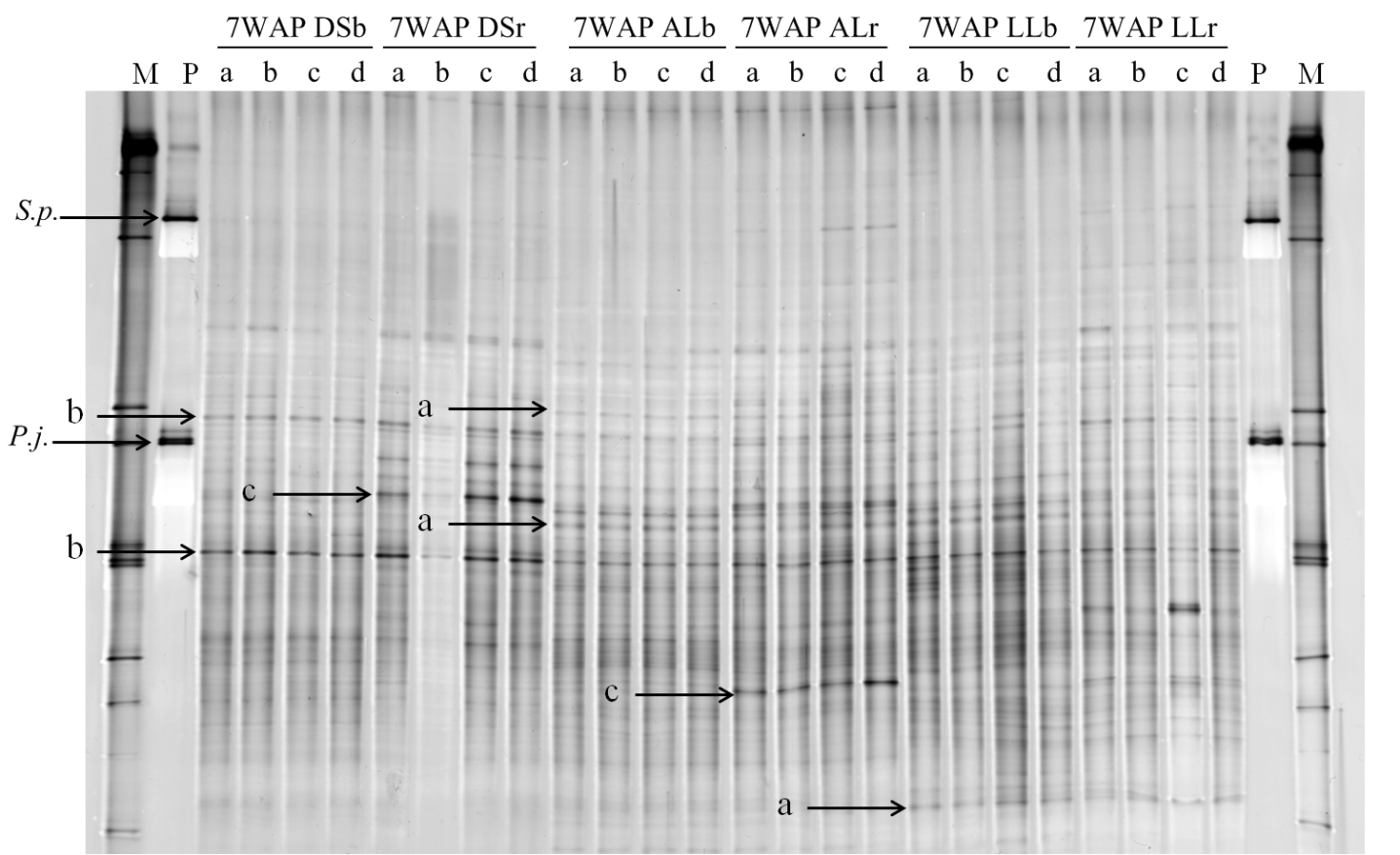


**Figure S5 | Dendrogram of bacterial community fingerprints three weeks after planting (3WAP).** UPGMA cluster analysis based on Pearson similarity matrices obtained by DGGE from samples taken 3WAP from DS, AL, and LL bulk soils (b) and the corresponding lettuce rhizosphere (r), a-d:4 replicates.

**
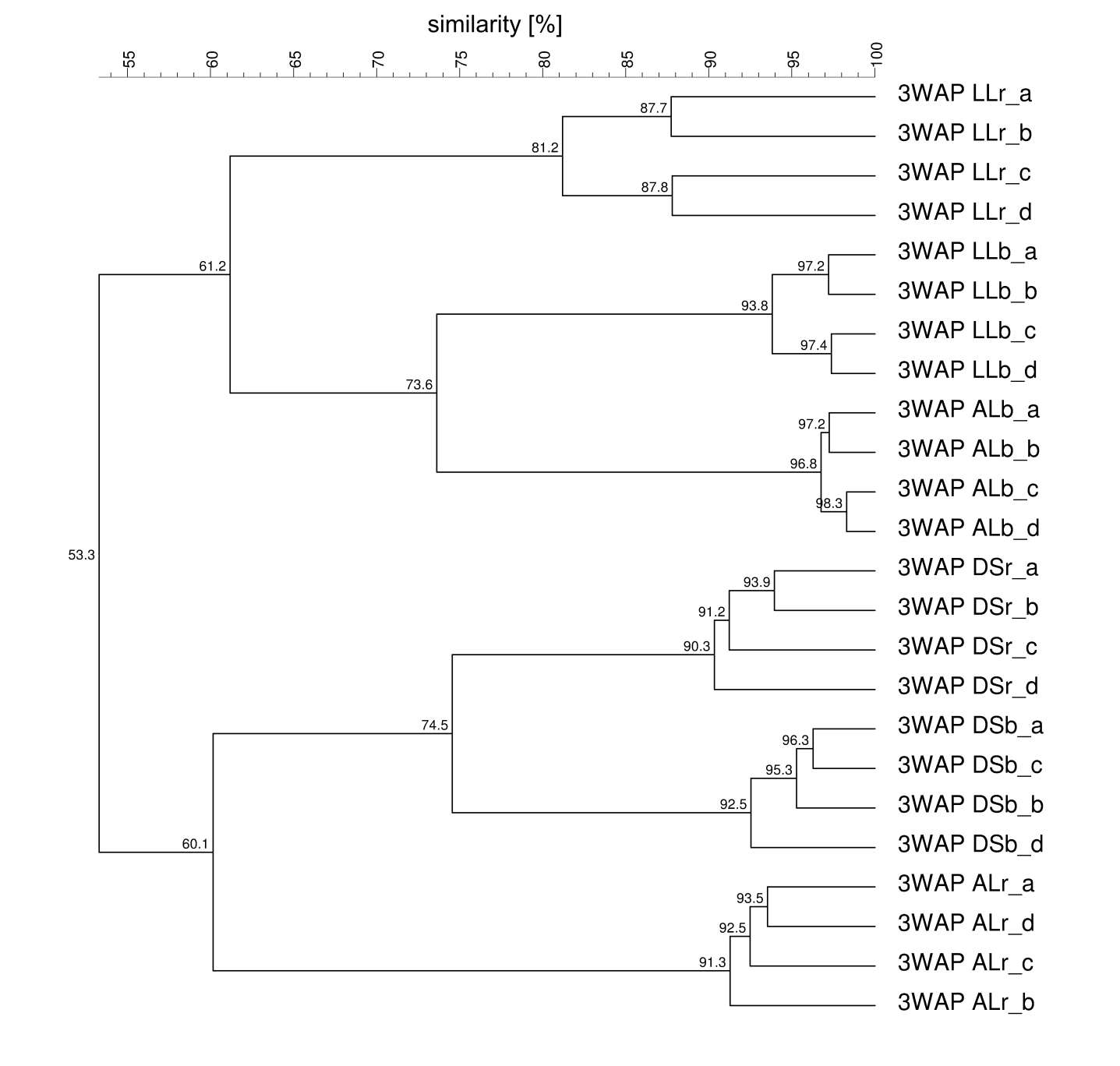
**

**Figure S6 | Dendrogram of bacterial community fingerprints seven weeks after planting (7WAP).** UPGMA cluster analysis based on Pearson similarity matrices obtained by DGGE from samples taken 7WAP from DS, AL, and LL bulk soils (b) and the corresponding lettuce rhizosphere (r), a-d:4 replicates .


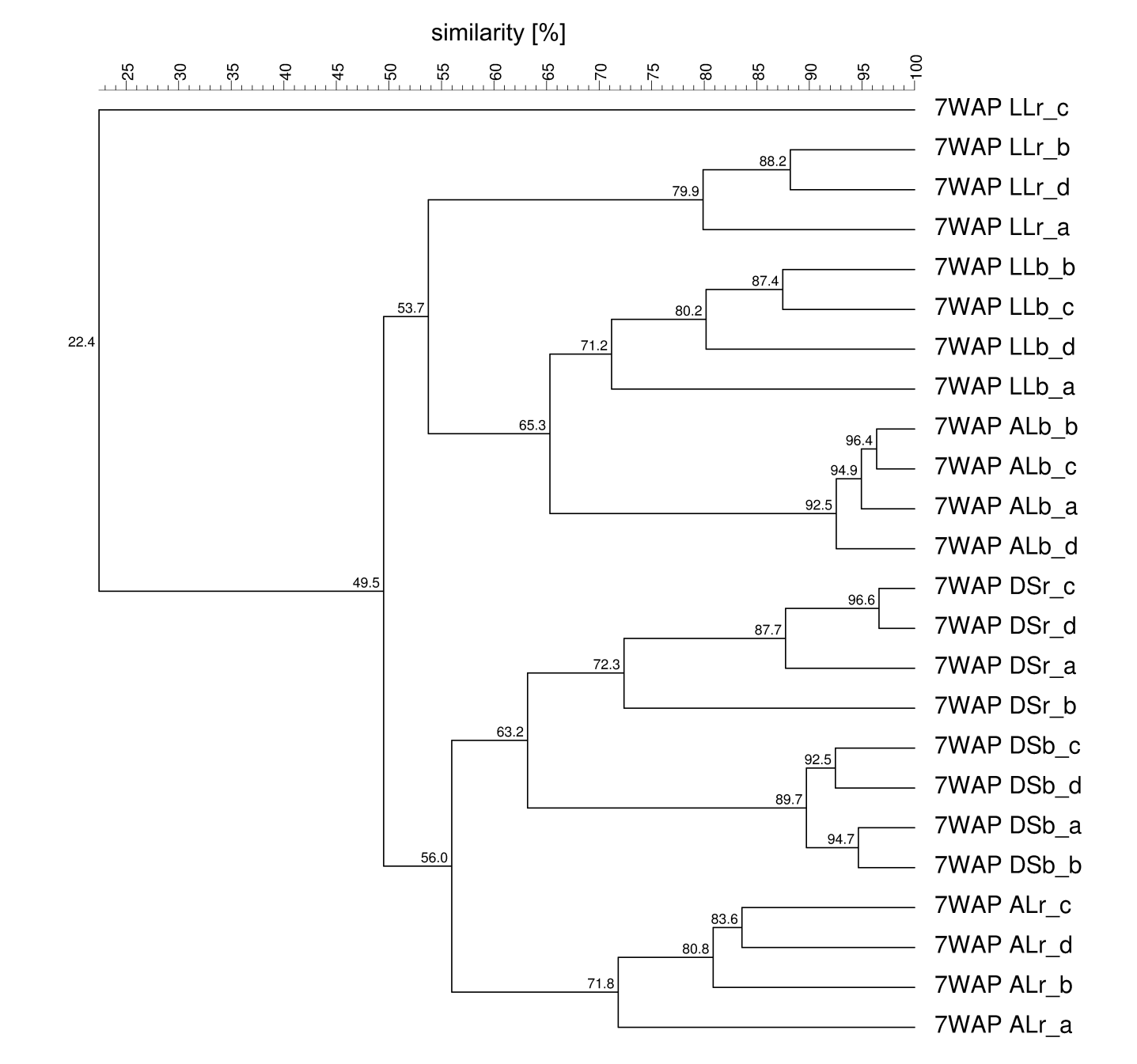


**Figure S7 | *Bacteria* DGGE fingerprint**. Samples were taken in the greenhouse before planting lettuce into the field (beforeP), three weeks after planting (3WAP), and seven weeks after planting (7WAP) from rhizosphere (r) of lettuce grown in DS, AL, or LL soil. M: Marker; letters a-d on top of lanes indicate the four replicates per treatment; e: dominant bands present 3WAP and 7WAP; f: bands present in all soil types and sampling times.


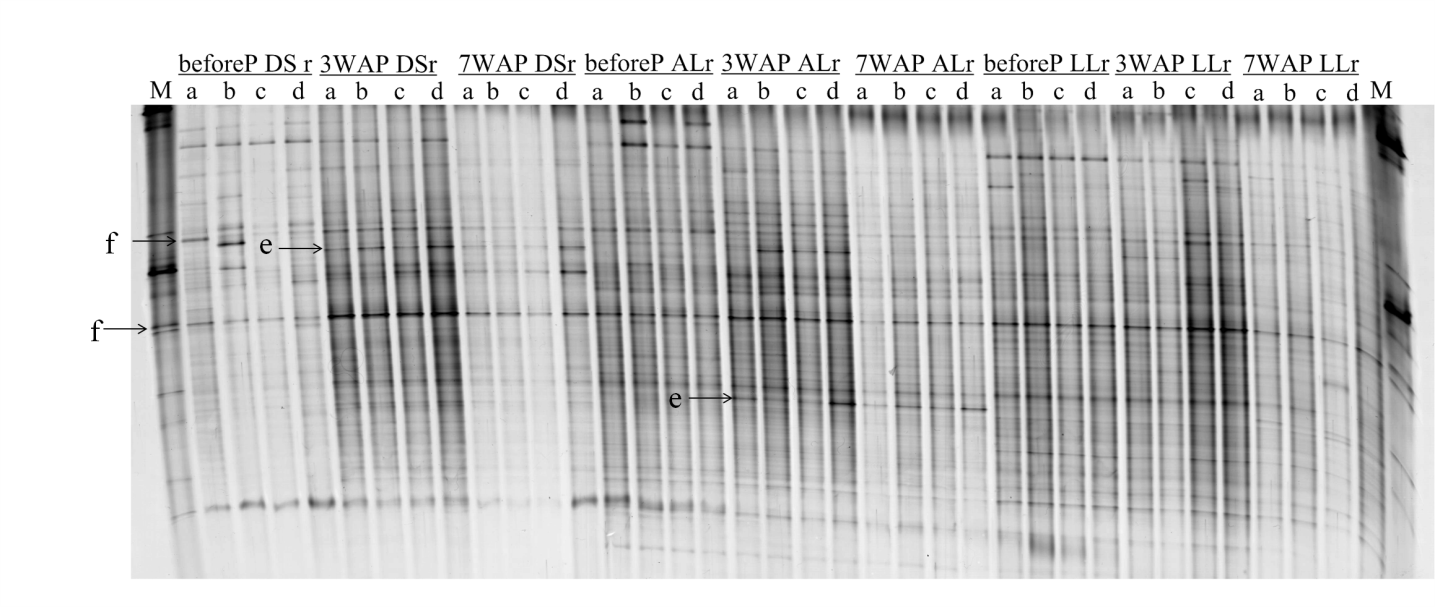

Supplement: Supplementary file 1 [file DataSheet1.DOCX]
